# Test characteristics of acute kidney injury biomarkers in animal models of sepsis

**DOI:** 10.1186/cc13567

**Published:** 2014-03-17

**Authors:** Z Peng, J Zhang, F Zhou, J Kellum

**Affiliations:** 1 University of Pittsburgh Medical Center, Pittsburgh, PA, USA

## Introduction

Approximately 50% of acute kidney injury (AKI) is associated with sepsis. Neutrophil gelatinase-associated lipocalin (NGAL) and cystatin C are the two most widely used biomarkers for AKI. However, these two markers are also affected by the systemic inflammatory response, and their diagnostic value in sepsis-induced AKI is disputed [1],[2]. Unlike clinical AKI, animal models can be used to explore single etiology. The purpose of this study is to examine the relationship between inflammatory mediators and biomarkers for AKI in a sepsis model in rats.

## Methods

Sepsis was induced by cecal ligation and puncture (CLP) in 60 adult SD rats and then observed for AKI and survival. Blood and urine samples were collected at baseline, and 18, 22, and 48 hours after CLP. AKI severity was assessed by RIFLE criteria (creatinine only). The associations between plasma IL-6 and plasma NGAL, plasma cystatin C, urine NGAL and urine cystatin C were analyzed. The area under the receiver-operator characteristic curves (AUC) was used to evaluate the diagnostic capability between severe AKI (RIFLE-I or RIFLE-F) and no AKI (includes RIFLE-R) for different biomarkers.

## Results

The changes of plasma NGAL, plasma cystatin C, urine NGAL and urine cystatin C with time were similar to the changes of plasma IL-6. However, only plasma NGAL levels were closely correlated with levels of plasma IL-6 (*R*^2 ^= 0.36, *P *< 0.05). The analysis for plasma cystatin C, urine NGAL and urine cystatin C at 22 hours for severe AKI showed AUCs of 0.78, 0.71 and 0.75 respectively (all *P *< 0.05), and the AUC for plasma NGAL was 0.62 (*P *= 0.11). There were no significant differences in plasma NGAL at 22 hours between severe AKI and no AKI (2,143.32 vs. 2,077.02 U/ml, *P *= 0.21).

## Conclusion

In this animal model of CLP sepsis, plasma NGAL levels were affected by the systemic inflammatory response, and did not discriminate for AKI. Urine NGAL, plasma cystatin C and urine cystatin C were able to differentiate severe AKI from no AKI in CLP sepsis.
